# Are Canadian Women Prepared for the Transition to Primary HPV Testing in Cervical Screening? A National Survey of Knowledge, Attitudes, and Beliefs

**DOI:** 10.3390/curroncol30070512

**Published:** 2023-07-24

**Authors:** Ben Haward, Ovidiu Tatar, Patricia Zhu, Gabrielle Griffin-Mathieu, Emily McBride, Jo Waller, Julia Brotherton, Aisha Lofters, Marie-Hélène Mayrand, Samara Perez, Zeev Rosberger

**Affiliations:** 1 Lady Davis Institute for Medical Research (LDI), Jewish General Hospital, Montreal, QC H3T 1E2, Canadazeev.rosberger@mcgill.ca (Z.R.); 2 Research Center, Centre Hospitalier de l’Université de Montréal (CRCHUM), Montreal, QC H2X 0A9, Canada; 3 Institute of Psychiatry, Psychology & Neuroscience, King’s College London, London SE5 8AF, UK; 4 Cancer Prevention Group, School of Cancer & Pharmaceutical Sciences, King’s College London, London SE1 9NH, UK; 5 Melbourne School of Population and Global Health, University of Melbourne, Melbourne, VI 3010, Australia; 6 Department of Family and Community Medicine, University of Toronto, Toronto, ON M5G 1V7, Canada; 7 Département d’Obstétrique-Gynécologie, Université de Montréal, Montreal, QC H3C 3J7, Canada; 8 Research Institute of the McGill University Health Centre (RI-MUHC), Montreal, QC H4A 3J1, Canada; 9 Department of Oncology, McGill University, Montreal, QC H4A 3T2, Canada; 10 Departments of Psychology and Psychiatry, McGill University, Montreal, QC H3A 1G1, Canada

**Keywords:** knowledge attitudes and beliefs, cervical cancer screening, HPV, HPV testing, women, web-based survey, self-sampling

## Abstract

As Canadian provinces and territories prepare to transition to HPV-based primary screening for cervical cancer, failure to identify and address potential barriers to screening could hinder program implementation. We examined screening-eligible Canadians’ attitudes towards and knowledge of cervical screening. A nationally representative sample of screening-eligible Canadians (*N* = 3724) completed a web-based survey in the summer of 2022. Oversampling ensured that half of the sample were underscreened for cervical cancer (>3 years since previous screening or never screened). The participants completed validated scales of cervical cancer, HPV, and HPV test knowledge and HPV test and self-sampling attitudes and beliefs. Between-group differences (underscreened vs. adequately screened) were calculated for scales and items using independent sample *t*-tests or chi-square tests. The underscreened participants (*n* = 1871) demonstrated significantly lower knowledge of cervical cancer, HPV, and the HPV test. The adequately screened participants (*n* = 1853) scored higher on the *Confidence* and *Worries* subscales of the HPV Test Attitudes and Beliefs Scale. The underscreened participants scored higher on the *Personal Barriers* and *Social Norms* subscales. The underscreened participants also endorsed greater *Autonomy* conferred by self-sampling. Our findings suggest important differential patterns of knowledge, attitudes, and beliefs between the underscreened and adequately screened Canadians. These findings highlight the need to develop targeted communication strategies and promote patient-centered, tailored approaches in cervical screening programs.

## 1. Introduction

For decades, cytology-based (i.e., Pap test) screening in Canada has resulted in a significant decline in cervical cancer rates. However, there remains an estimated 1450 diagnoses (estimated age-standardized incidence rate in 2022: 7.5 ) and 380 deaths from cervical cancer in Canada annually (estimated age-standardized mortality rate in 2022: 1.8 [1]) [2]. The most recent estimates of screening participation in Canada are estimated at 60 and 75%, depending on province and age [3], falling below the Canadian Partnership Against Cancer’s (CPAC) goal of screening 90% of all eligible individuals and 80% of individuals within any identifiable group [4]. Of individuals diagnosed with cervical cancer in Canada, an estimated 37% have not been screened in the prior 5-year period or have never been screened [3]. In addition, ethnic, linguistic, gender, and sexual minority groups, as well as those living in remote and rural areas, represent a disproportionate number of underscreened individuals and, subsequently, cervical cancer diagnoses in Canada [4].

Cytology-based screening has limited sensitivity and often leads to missed cervical lesions. This issue could be amplified given lower base rates of abnormalities due to increased HPV vaccination [5]. In contrast, HPV-based screening has greater sensitivity in detecting pre-cancerous lesions and allows for longer intervals between screening [6,7]. Multiple health organizations, including the World Health Organization (WHO) and CPAC, now recommend HPV testing as the primary method for cervical screening [4,7,8,9]. HPV testing also allows for self-sampling, in which an individual can obtain a sample themselves using a vaginal swab included in a self-sampling kit either at home or in a clinic, rather than undergoing health care provider (HCP)-administered collection. This approach presents a promising avenue for those facing issues of accessibility, (e.g., living in remote/rural regions and/or having no regular primary care practitioner) and those finding screening by a provider to be potentially uncomfortable, embarrassing, stigmatizing, or anxiety provoking [10,11].

Experience from other countries (e.g., Australia and Wales) has shown that transitioning to HPV testing as the primary screening method without adequate and anticipatory population-wide preparation can result in confusion, misinformation, and mistrust [12,13]. Similar reactions can be expected in Canada unless effective information and messaging are introduced prior to implementation. By identifying existing knowledge gaps and belief/attitudinal differences between those who are underscreened and those who are adequately screened, targeted messaging can be proactively developed and promoted to both ensure increased uptake and continued engagement in recommended screening practices.

The current study was conducted as part of a multi-phase project aimed at understanding Canadian women’s preparedness for the transition from cytology to HPV test-based primary screening to prevent cervical cancer. The present study objectives were: (1) to describe HPV, the HPV test, and cervical cancer knowledge and attitudes regarding HPV testing and self-sampling in adequately and underscreened women and (2) to estimate differences in knowledge and attitudes and beliefs between the two groups (see study 2, objective 1 in our published protocol [14]).

## 2. Materials and Methods

### 2.1. Study Design and Participants

A detailed description of the study methodology can be found elsewhere [14]. In brief, screening-eligible Canadians were invited to complete a web-based survey in either English or French in August−September 2022. The inclusion criteria were having a cervix and being aged 21–70 (the youngest and oldest ages to be eligible for screening in Canada). The exclusion criteria were having a previous diagnosis of cervical cancer or not having a cervix (i.e., previous hysterectomy). Oversampling was used so that half of the sample were underscreened for cervical cancer (>3 years or never had a Pap test based on self-reporting) and the other were adequately screened (<3 years since previous screening). Census-based quotas were applied for age, province, primary language, household income, and rural/urban residence to reinforce sample representativeness. The study received ethical approval from the Research Ethics Broad of the Integrated Health and Social Services University Network (CIUSSS) West-Central Montreal (Project ID: 2022–2960).

### 2.2. Procedure

The participants provided information regarding sociodemographics, relevant health behaviours, and cervical screening in either English or French. At various points during the survey, the participants were shown informative statements which provided minimal and neutral information to orient them towards the items that followed. For example, before completing items related to self-sampling, the participants were informed about the availability of and procedures to conduct self-sampling with an accompanying infographic demonstrating the procedure, since this screening option is currently unknown to most Canadians. These informative statements are available in the published protocol [14].

### 2.3. Measures

#### 2.3.1. Screening History

The participants were asked when they last received a Pap test with the following response options: within the last year; within the last 1 to 3 years; over 3 years ago; or never. Those who responded as having received screening within the past year or in the previous 1 to 3 years were classified as *adequately screened* (considering 2–3-year intervals recommended in most Canadian provinces [15]), and those who had never received screening or not in the past 3 years were labelled *underscreened*. 

#### 2.3.2. Knowledge Scales

The participants completed three scales measuring knowledge of cervical screening-related topics. The Cervical Cancer Knowledge Scale (CCKS) and HPV Testing Knowledge Scale (HTKS) were developed by our team and each contain eight items [16]. HPV knowledge was measured using the HPV General Knowledge Scale developed by Perez et al. [17], which contains 23 items and expands upon the scale by Waller et al. [18]. All items were answered with “*true*”, “*false*”, or “*I don’t know*”. The response option of “*I don’t know*” was included to counteract errors due to random responses or guessing, and such responses were categorized as incorrect for the analyses. The Cronbach’s α values for the present study were as follows: CCKS (α = 0.78); HTKS (α = 0.73); HPV General Knowledge Scale (α = 0.90).

#### 2.3.3. Attitudes and Beliefs Scales

The participants completed the HPV Testing Attitudes and Beliefs Scale (HTABS) and HPV Self-Sampling Attitudes and Beliefs Scale (HSABS) as developed and validated by our team [19]. The HTABS contains four factors: *Personal Barriers* (7 items, e.g., “I would be embarrassed to get tested for HPV because it is a sexually transmitted infection”), *Social Norms* (4 items, e.g., “my friends’ opinion about getting the HPV test would be important to me”), *Confidence* (6 items, e.g., “having the HPV test would be a good way to identify problems before they become cancer ”), and *Worries* (3 items, e.g., “I would be worried about getting tested with the HPV test less often than every 3 years”). The HSABs contains two factors: *Concerns* (4 items, e.g., “if I did HPV self-sampling, I could harm myself”) and *Autonomy* (3 items, e.g., “if I did HPV self-sampling, I would be more in control of my body”). The participants responded to the attitudes and beliefs items on a seven-point Likert scale from “*strongly disagree*” to “*strongly agree*”.

### 2.4. Analyses

For each knowledge item, the proportion of participants who correctly answered the item was calculated. Total knowledge scale scores were calculated as the total number of correct responses across all relevant items. For the attitudes and beliefs scales, mean scores for each item and subscale (calculated as the mean score across all relevant items) were calculated.

Chi-square tests of independence were used to examine differences on individual knowledge items between adequately screened and underscreened participants, and for significant differences, we calculated the effect size (Cohen’s h). For continuous data (i.e., individual attitudes and beliefs items and the attitudes and beliefs subscales and knowledge scales total scores), we used independent sample *t*-tests to evaluate between-group differences and calculated the effect size (Cohen’s d). Bonferroni corrections were used to adjust significance levels for multiple comparisons according to the number of items within a given scale or subscale. We used the benchmarks defined by Cohen [20] to interpret effect sizes (very small < 0.2, small 0.2 to 0.49, medium 0.5 to 0.79, and large ≥ 0.8). To evaluate between-item differences, we calculated the 95% confidence intervals for proportions (knowledge items) and means (attitudes and beliefs items) using bootstrapping (1000 replicates). We conducted these analyses for each knowledge scale and attitudes and beliefs subscale and separately for underscreened and adequately screened participants. We used SPSS version 24.0 [21] and R 4.2.2 [22] statistical software.

## 3. Results

### 3.1. Participants

In total, 4082 participants completed the survey. After data cleaning methods were applied (e.g., attention-check items and outlying response times [14]), 3724 observations were retained for analysis. Of these, 1871 were adequately screened and 1853 were underscreened. The sociodemographic characteristics of adequately screened and underscreened participants are reported in Table 1. While significant sociodemographic differences were observed between groups, the effect sizes were very small to small (see Table 1).

### 3.2. Cervical Cancer Knowledge

**Figure 1:**
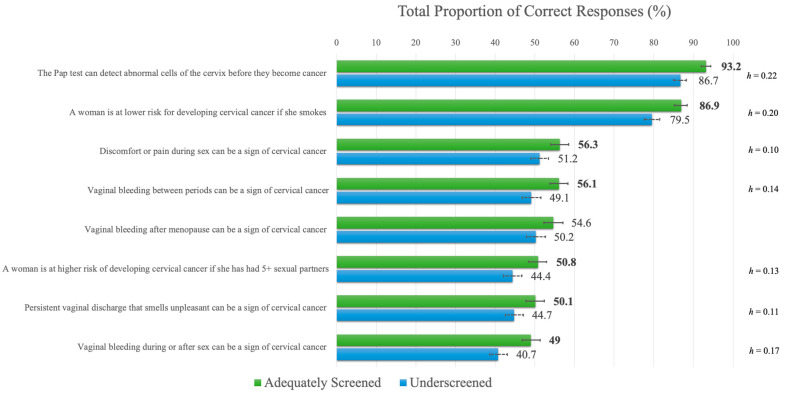
Item-level differences for the Cervical Cancer Knowledge Scale. **Note.** Significant *p*-values, adjusted for multiple comparisons using Bonferroni corrections (i.e., *p* < 0.006), are provided in boldface. Error bars indicate 95% confidence intervals. Effect sizes (Cohen’s *h*) are provided for significant item-level differences.

The mean (*M*) score for the full sample for the CCKS was 4.72 out of a possible 8 (standard deviation (*SD*) = 2.26). Adequately screened participants (*M* = 4.97, *SD* = 2.19) had significantly higher scores than underscreened participants (*M* = 4.46, *SD* = 2.30), t(3722) = 6.870, *p* < 0.001, *d* = 0.23. Adequately screened participants had significantly higher correct responses on seven of the eight items of the CCKS (see Figure 1), although the effect sizes were very small. Examination of item-level differences demonstrated significantly higher knowledge in both groups of the item “The Pap test can detect abnormal cells of the cervix before they become cancer” (*true*) versus all other items. This item and the item “A woman is at lower risk for developing cervical cancer if she smokes” (*false*) significantly exceeded knowledge of the remaining six items, among which knowledge levels were largely indistinct (see Figure 1). More detailed results are available in Supplementary Material S1.

### 3.3. HPV Testing Knowledge

**Figure 2:**
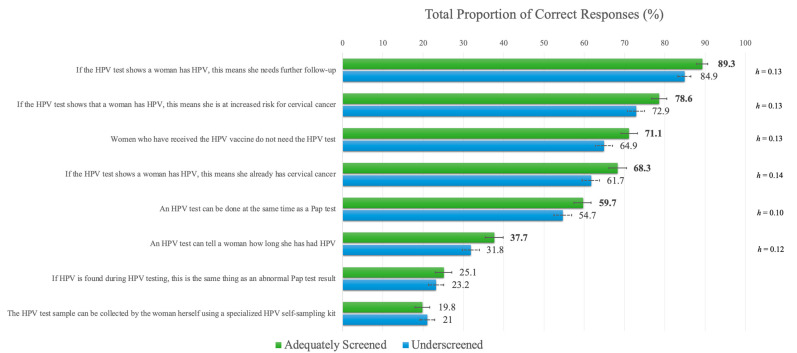
Item-level differences for the HPV Testing Knowledge Scale. **Note.** Significant *p*-values, adjusted for multiple comparisons using Bonferroni corrections (i.e., *p* < 0.006), are provided in boldface. Error bars indicate 95% confidence intervals. Effect sizes (Cohen’s *h*) are provided for significant item-level differences.

The mean score for the full sample for the HTKS was 4.32 out of a possible 8 (*SD* = 1.93). Adequately screened participants (*M* =4.49, *SD* = 1.87) had significantly higher mean scores than underscreened participants (*M* = 4.15, *SD* = 1.99), t(3722) = 5.421, *p* < 0.001, *d* = 0.18. Regarding item-level differences, adequately screened participants had significantly higher correct responses on six of the eight items of the HTKS (see Figure 2), although the effect sizes were very small. Item-level analysis demonstrated significantly higher knowledge for the item “*If the HPV test shows a woman has HPV, this means she needs further follow-up*” in both groups versus all other items. Two items had distinctly lower proportions of correct responses versus the other six items, “*If HPV is found during HPV testing, this is the same as an abnormal Pap test result*” (*false*) and “*The HPV test sample can be collected by the woman herself using a specialized HPV self-sampling kit*”. Adequately screened participants had significantly higher knowledge of the item pertaining to the difference between HPV and Pap test results versus the item about the availability of self-sampling, but this difference was not observed for the underscreened participants. More detailed results are available in Supplementary Material S1.

### 3.4. HPV Knowledge

**Figure 3:**
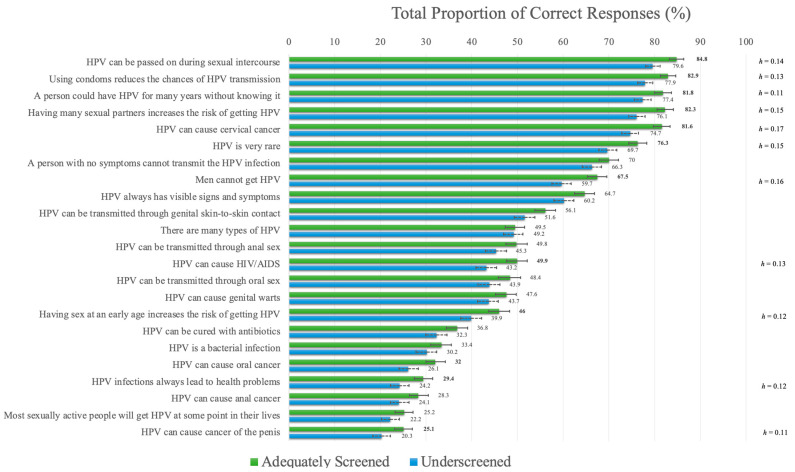
Item-level differences for the HPV General Knowledge Scale. **Note.** Significant *p*-values, adjusted for multiple comparisons using Bonferroni corrections (i.e., *p* < 0.002), are provided in boldface. Error bars indicate 95% confidence intervals. Effect sizes (Cohen’s *h*) are provided for significant item-level differences.

The mean score for the full sample for the HPV Knowledge Scale was 11.94 out of a possible 23 (*SD* = 5.44). Adequately screened participants (*M* =12.49, *SD* = 5.34) had significantly higher scores than underscreened participants (*M* = 11.38, *SD* = 5.49), t(3722) = 6.265, *p* < 0.001, *d* = 0.21. Adequately screened participants had significantly higher correct responses on twelve of the twenty-three items of the HPV Knowledge Scale (see Figure 3), although the effect sizes were very small. Item-level differences demonstrated five items with similarly high proportions of correct responses among adequately screened participants and four items among underscreened participants. These items pertained to knowledge that HPV is sexually transmitted, condoms reduce HPV transmission, HPV can be present for many years without symptoms, increased sexual partners are associated with HPV risk, and HPV’s causal role in cervical cancer. Seven items displayed similarly low proportions of correct responses in both groups, which included those related to HPV causing oral, anal and penile cancers, most people having a lifetime HPV infection, and HPV always leading to health problems. More detailed results are available in Supplementary Material S1.

### 3.5. HPV Testing Attitudes and Beliefs 

**Figure 4:**
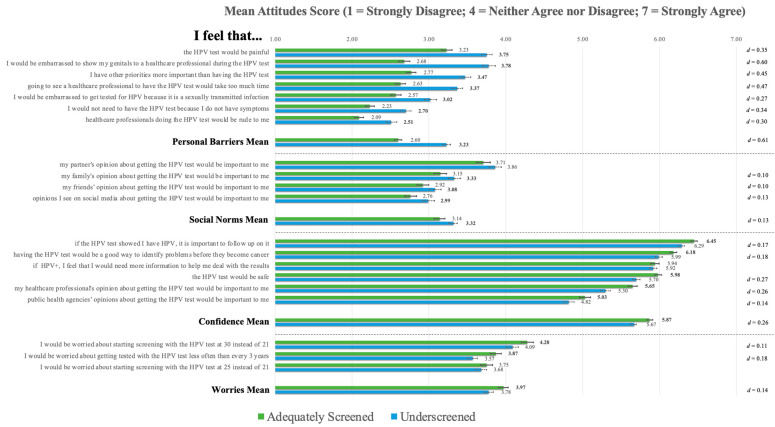
Item-level differences for the HPV Testing Attitudes and Beliefs Scale. **Note.** Significant *p*-values, adjusted for multiple comparisons using Bonferroni corrections (i.e., *p_barriers_* < 0.007; *p_norms_* < 0.012; *p_confidence_* < 0.008; *p_worries_* < 0.016), are provided in boldface. Error bars indicate 95% confidence intervals. Effect sizes (Cohen’s *d*) are provided for significant item-level differences.

Results for each subscale of the HTABS are presented below. All item-level scores and differences are available in Figure 4. More detailed results are available in Supplementary Material S1 .

#### 3.5.1. Personal Barriers

The underscreened (*M* = 3.23, *SD* = 1.05) participants had significantly higher mean scores on the *Personal Barriers* subscale of the HTABS compared to the adequately screened participants (*M* = 2.60, *SD* = 1.02), t(3722) = 18.530, *p* < 0.001, *d* = 0.61, and had significantly higher scores across all seven items, suggesting that participants in this group have greater and more multifaceted perceived barriers to screening. In the item-level differences, among the adequately screened participants, the highest agreement was observed for the item “the HPV test would be painful”, whereas the underscreened participants demonstrated similarly high agreement between this item and “I would be embarrassed to show my genitals to a healthcare professional during the HPV test”. One item had distinctly lower agreement versus all others in both groups, “healthcare professionals doing the HPV test would be rude to me”.

#### 3.5.2. Social Norms

The underscreened participants (*M* = 3.31, *SD* = 1.36) had significantly higher mean scores on the *Social Norms* subscale compared to the adequately screened participants (M = 3.14, SD = 1.43), although the effect was very small, t(3722) = 3.935, *p* < 0.001, *d* = 0.13. The underscreened participants also had significantly higher scores on three of the four items of this subscale. The item “my partner’s opinion about getting the HPV test would be important to me” had distinctly higher agreement in both groups versus the other social norm items, with no significant difference observed between the groups. A similar item pertaining to one’s family had higher agreement than two items relating to the importance of the opinion of friends and social media in HPV test decision-making.

#### 3.5.3. Confidence

The adequately screened participants (*M* = 5.87, *SD* = 0.78) had significantly higher scores on the *Confidence* subscale compared to the underscreened participants (*M* = 5.67, *SD* = 0.83), t(3722) = 7.787, *p* < 0.001, *d* = 0.26) and across five of the six items, which could indicate greater general trust in HPV testing. Agreement was relatively high on this subscale, with scores on all items exceeding the item-level scores on all other HTABs subscales. The item “if the HPV test showed I had HPV, it is important to follow up on it” had distinctly higher agreement in both groups versus all other items. Contrastingly, an item pertaining to the importance of public health agencies’ recommendations for HPV testing had significantly lower agreement in both groups than any other item.

#### 3.5.4. Worries

The adequately screened participants (*M* = 3.97, *SD* = 1.37) had significantly higher scores than the underscreened participants on the *Worries* subscale (*M* = 3.78, *SD* = 1.23), although the effect was very small, t(3722) = 4.392, *p* < 0.001, *d* = 0.14. This could reflect greater concerns about increased screening intervals and ages in this group, which the items in this subscale examined. However, the effect size was very small. The adequately screened participants had higher scores on two of the three items on this subscale. The participants in both groups expressed distinctly high agreement for the item “I would be worried about starting screening with the HPV test at 30 years old instead of 21 years old” versus the two remaining items, between which no difference in agreement was observed.

### 3.6. HPV Self-Sampling Attitudes and Beliefs

**Figure 5:**
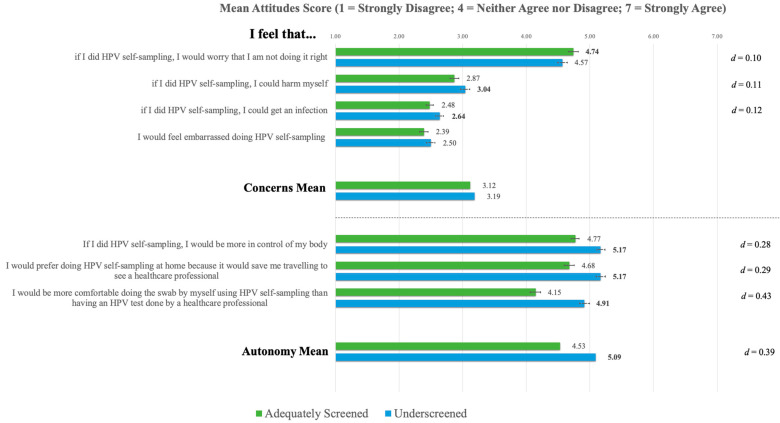
Item-level differences for the HPV Self-Sampling Attitudes and Beliefs Scale. **Note.** Significant *p*-values, adjusted for multiple comparisons using Bonferroni corrections (i.e., *p_concerns_* < 0.012; *p_autonomy_* < 0.016), are provided in boldface. Error bars indicate 95% confidence intervals. Effect sizes (Cohen’s *d*) are provided for significant item-level differences.

Results for each subscale of the HSABS are presented below. All item-level scores and differences are available in Figure 5.

#### 3.6.1. Concerns

There was no significant difference between the underscreened and adequately screened participants on the *Concerns* subscale of the HSABS, t(3722) = 1.707, *p* = 0.088. However, significant differences were observed across three of the four items in this subscale (see Figure 5), with adequately screened participants indicating higher worry about carrying out self-sampling incorrectly and underscreened participants expressing greater concern about harming oneself or getting an infection. However, the effect sizes for these differences were very small. The item “if I did self-sampling, I would worry that I am not doing it right” had significantly higher agreement in both groups versus all other items, and the item “if I did self-sampling, I could harm myself” was distinctly higher than the other two remaining items. Between the items pertaining to the potential for infection and embarrassment due to self-sampling, no difference in agreement was observed. 

#### 3.6.2. Autonomy

The underscreened participants (*M* = 5.09, *SD* = 1.37) had significantly higher mean scores than the adequately screened participants (*M* = 4.53, *SD* = 1.46) on the *Autonomy* subscale, t(3722) = 11.883, *p* < 0.001, *d* = 0.39 and across all three items. Item-level analysis indicated that agreement was similarly high in both groups between two items pertaining to agreement that self-sampling confers greater bodily control and would save travelling time and distinctly higher than the item “I would be more comfortable doing the swab by myself using HPV self-sampling than having an HPV test done by a healthcare professional”.

## 4. Discussion

The present study examined the knowledge, attitudes, and beliefs of cervical screening-eligible Canadians in the context of HPV-based screening implementation. Our results indicated that, in general, the underscreened participants had lower knowledge of cervical cancer, HPV testing, and HPV. The examination of attitudes and beliefs indicated a unique pattern of perceptions between groups and highlighted possible barriers and facilitators to HPV-based screening acceptance for adequately and underscreened individuals.

### 4.1. Knowledge

Across all three knowledge scales that measure cervical cancer, HPV, and HPV testing knowledge, the underscreened participants had significantly lower knowledge, although the effect sizes were small. For cervical cancer knowledge, participants across both groups demonstrated high knowledge of the purpose of the Pap test in identifying pre-cancers, contrasting with findings from a study in the U.S. by Kasting et al. [24], who found that only 26% of participants understood this. Importantly, their sample consisted solely of ethnic minority groups, and such knowledge gaps might still be present in similar subpopulations in Canada and would warrant further investigation. Knowledge of cervical cancer symptoms, such as vaginal bleeding and discharge, was poor in both the adequately and underscreened group. Low knowledge about the early signs and symptoms of cervical cancer is concerning, especially in underscreened women who may be delayed in recognizing symptoms and seeking care [25].

Regarding general HPV knowledge, knowledge that HPV causes cervical cancer was generally high (78.2% correct; “HPV can cause cervical cancer” true) but was significantly higher in adequately screened (81.6%) versus underscreened (74.7%) women. Consistent with this, the results from the HPV Testing Knowledge Scale confirmed that most participants (75.8%) understood that an HPV-positive test result indicated an increased cervical cancer risk. However, knowledge of the high lifetime incidence of HPV and the likelihood of sequalae following infection was low, suggesting that, while participants understood HPV’s relationship with cervical cancer, they were less knowledgeable about the probability of becoming infected or developing HPV-related diseases. Importantly, knowledge of HPV causing genital warts and oral, anal and penile cancers was also low and similar to the results observed by Thompson et al. [26], in which only 36.1% of U.S. participants were aware of HPV causing non-cervical cancers. Preti et al. [27] found that of those being treated for high-grade cervical lesions (CIN 2 or 3), there is an estimated 2.2 times elevated risk (standardized incidence ratio [SIR]) for other HPV-related cancers, including an SIR of 8.5 for cancers of the oropharynx. Our findings highlight the importance of increasing awareness of and education about [28] other HPV-related cancers (including oropharyngeal cancers) to support both participation in HPV vaccination and screening programs and encourage further screening in the case of HPV+ cervical screening results [29]. Knowledge of HPV self-sampling was limited (20.4% correct), demonstrating that significant engagement from public health authorities and clinicians is needed to inform women about this method of screening.

### 4.2. HPV Testing Attitudes and Beliefs

Scores on the *Confidence* subscale of the HTABS were higher than for all other subscales, showing the importance of this dimension, independent of screening status. This is consistent with findings suggesting that women’s perceptions of HPV testing are not only related to the perceived performance of the test itself but also to participants’ understanding of the rationale for using it (e.g., the meaning of a positive result and knowledge of HPV’s relationship with cervical cancer) [30,31,32]. Interestingly, in analyzing the comments of an online survey opposing changes to the Australian cervical cancer program, Obermair et al. [13] found that most concerns were associated with increases to screening intervals and later ages of screening initiation rather than the HPV test itself. Higher scores among adequately screened women on the *Worries* subscale of the HTABS, which contains items pertaining to increased screening ages and intervals, support this observation. Like those individuals who supported the petition in Australia to not shift to HPV testing, adequately screened Canadians might be more likely to be worried about going through testing with the HPV test less often than every 3 years considering that, unlike the underscreened participants, it would alter the screening regimen to which they are already accustomed. Within the Worries subscale, the participants in both groups were most concerned about the age of initial screening being adjusted to 30 rather than 25, confirming findings from a recent nationwide survey showing low preference for screening initiation at age 30 [33]. Considering evidence from the organized cervical screening program in the Netherlands that HPV testing could be safely introduced at 30 years using a screening interval of 5 years [34], public health authorities should prioritize communications addressing concerns about the starting age of screening and longer screening intervals compared to Pap, which will likely be mirrored in Canada.

The consistent between-group differences on the *Personal Barriers* subscale emphasize the importance of addressing psychological (e.g., embarrassment) and practical (e.g., time) barriers and screening-discordant beliefs (e.g., only needing to screen if symptoms are present). Such concerns have been highlighted in previous reviews, both of cervical screening generally [35,36,37,38] and HPV testing specifically [30], and are often driven by cultural concerns. Increasingly, studies have emphasized the importance of patient engagement and co-designed programs, particularly among minority groups [39]. By creating culturally safe, community-focused program designs and communications, Canadian programs could address many of these barriers to screening in groups that are most at risk of developing cervical cancer. Concurrent with our findings, studies have also identified a lack of time [37,40] and low priority for screening [41] as barriers to screening for underscreened groups. Higher scores on the item “I would be embarrassed to get tested for HPV because it is a sexually transmitted infection” among the underscreened women are consistent with the findings of systematic reviews by Tatar et al. [30] and Nothacker et al. [31], which identified stigma as a barrier to HPV testing. Without adequate education and clear recommendations from health authorities, stigma not only poses challenges to screening uptake but also contributes psychosocial distress after a positive test result [42] and increases the likelihood of failing to maintain preventive behaviours like screening [43].

Higher scores on the *Social Norms* subscale of the HTABS in the underscreened participants could suggest the importance of designing interventions to promote screening-positive social norms within populations with low levels of screening. For example, a study by Knops-Dullens et al. [44] in the Netherlands found that screening attendees were more likely to report having positive role models for screening and perceived greater social norms to attend screening. Our study identified the opinion of partners as distinctly important in decision-making regarding HPV testing. Studies have demonstrated partner’s views as barriers to HPV-based screening, particularly considering the sexual implications of a positive HPV test [30]. However, there might also be an opportunity to foster screening engagement by promoting discussions with partners and leveraging communications with males to encourage such conversations. While endorsement of opinions from social media being important in decision-making about HPV testing was generally lower among both groups versus other social influences, higher scores among the underscreened participants highlight the importance of social media as a tool to reach these populations and the need to counteract possible online misinformation and disinformation that might discourage screening [45]. An examination of social network posts related to gynecological cancers in China by Chen et al. [46] demonstrated that upwards of 30% of posts included misinformation, underlining the critical importance of identifying and countering false understandings of HPV-based screening in addition to providing accurate information.

### 4.3. HPV Self-Sampling Attitudes and Beliefs

A systematic review and meta-analysis by Virtanen et al. [47] examining the self-sampling experiences of non-attendees to cervical screening showed that independence from healthcare settings and providers, both due to convenience and comfort, are advantages of self-sampling for this group. Our findings support this conclusion, with those who were underscreened reporting greater autonomy conferred by self-sampling, across items examining perceptions of bodily control, independence from an HCP, and convenience due to reduced travel. These findings align with investigations in Canada examining self-sampling in typically underscreened groups. A study by Racey and Gesink [48], in which Canadian women living in rural Ontario discussed self-sampling in focus groups, observed that women found that self-sampling addressed logistical barriers including limiting travel and time commitments associated with cervical screening and procedural barriers including embarrassment from clinician-administered sampling. Similarly, Zehbe et al. [49] found in a sample of First Nations women from Ontario that 67% would prefer self-sampling versus clinician-administered screening. Globally, studies have noted that self-sampling is often preferable for gender-diverse and sexual minority individuals for this reason [50,51].

Item-level differences on the *Concerns* subscale of the HSABS indicate a unique pattern of attitudes between adequately screened and underscreened participants. The underscreened participants typically had higher scores on items measuring harm (i.e., pain and infection) resulting from self-sampling, whereas adequately screened participants demonstrated greater concern about conducting sampling incorrectly (i.e., “if I did HPV self-sampling, I would worry that I am not doing it right”). These results suggest contrasting barriers to self-sampling participation between these groups and support the need to reassure those who have more limited experience with screening about the safety of self-sampling. These results align with a recently published Canadian study showing that adequately screened Canadians typically have a low preference for self-sampling compared to alternative screening methods [33]. Scores for the item concerning self-sampling being carried out incorrectly were higher than the items considering harm, infection, and embarrassment. This is consistent with extant findings that suggest fears about sample reliability and the collection procedure are the primary concerns of individuals using self-sampling [10,48,52]. As it seems likely that self-sampling will be offered in Canadian screening programs, communications should emphasize the similar fidelity of self-sampling and clinician-administered screening [53]. Attention must also be given to ensuring that instructions accompanying self-sampling kits are clear and accessible and that responsive and reciprocal communication is available should concerns arise [10,54].

### 4.4. Study Strengths and Limitations

This Canada-wide investigation of knowledge, attitudes, and beliefs towards HPV-based cervical screening is critically important given imminent changes to screening programs in multiple jurisdictions. The use of a large sample of underscreened and adequately screened women enabled accurate between-group comparisons to produce findings which can inform targeted communication and education strategies. However, the use of self-reported screening status could limit the applicability of our findings, given difficulties recalling past screenings. Future studies should consider using screening registries to identify screening histories. The use of validated scales, which were previously tested among Canadian women for the purpose of addressing implementation challenges for HPV-based screening [14], ensure the reliability and applicability of our findings. While the use of a web-based survey enabled nationwide recruitment, it may have also prevented the recruitment of digitally excluded populations. Further research is needed to confirm and compare these findings with populations that require specific, participatory action research in the context of cervical screening implementation (e.g., First Nations, Inuit, and Métis) or have small population-level representation (e.g., gender diverse identities).

## 5. Conclusions

The shift to HPV-based cervical screening in Canada provides an important opportunity to continue to reduce the burden of cervical cancer and reduce disparities in screening, diagnosis, and treatment. Our results support concerns from other countries’ experiences that progress cannot be made without adequate consultation and communication with the screening-eligible population. By conducting a population-wide and large web-based survey, this study illuminates the knowledge and attitudes about cervical screening that may be implicated in the acceptance and uptake of HPV-based screening in Canada. Our findings also indicate that successful screening programs should avoid a “one-size-fits-all” approach, both in their communication strategies and program design. For underscreened women, providing targeted education about HPV, testing, and cervical cancer, and addressing barriers to screening (including concerns about embarrassment and HPV-related stigma) will be critical. Including self-sampling in organized cervical screening programs could increase screening uptake in this group and communications should emphasize the personal choice (autonomy) and the comfort that this method provides. For adequately screened women, there is a need to address worries about increased screening intervals and later ages of initiation associated with HPV testing.

Successful implementation of primary HPV-based cervical screening, which will progress Canada’s goals for cervical cancer elimination, will require buy-in at all levels. Screening-eligible Canadians are key stakeholders in cervical screening and provincial governments, federal agencies, and organizations involved in cancer recommendations, care, and prevention, should continually consider their knowledge, attitudes and beliefs as changes are implemented. By investigating Canadians’ current perceptions of HPV-based cervical screening, this study provides a timely investigation which can aid in aligning public health strategies to address population-level knowledge gaps and attitudinal barriers.

## Figures and Tables

**Table 1:** Sample characteristics.

	Full Sample (*N* = 3724)	Adequately Screened (*n* = 1871)	Underscreened (*n* = 1853)	*p*-Value (Effect Size) ^1^
**Age (years), M (SD)**	44.97 (14.73)	46.44 (13.9)	43.50 (15.33)	<0.001 (*d* = 0.20)
**Region, *n* (%) ^2^**				<0.001
Western and Territories	1182 (31.7)	615 (32.9)	567 (30.6)	ns
Ontario	1453 (39.0)	759 (40.6)	694 (37.5)	ns
Quebec	826 (22.2)	352 (18.8)	474 (25.6)	*h* = 0.16
Atlantic	263 (7.1)	145 (7.7)	118 (6.4)	ns
**Area, *n* (%)**				0.954
Rural	743 (20.0)	374 (20.0)	369 (19.9)	–
Urban	2981 (80.0)	1497 (80.0)	1484 (80.1)	
**Ethnicity ^3^, n (%)**				<0.001
North American Indigenous	123 (3.3)	67 (3.6)	56 (3.0)	ns
North American-Other	1653 (44.4)	840 (44.9)	813 (43.9)	ns
European	1120 (30.1)	618 (33.0)	502 (27.1)	*h* = 0.13
Asian	529 (14.2)	203 (10.8)	326 (17.6)	*h* = 0.19
Other ^4^	299 (8.0)	143 (7.6)	156 (8.4)	ns
**Visible minority, *n* (%)**				<0.001
Yes	741 (19.9)	306 (16.4)	435 (23.5)	*h* = 0.18
No	2983 (80.1)	1565 (83.6)	1418 (76.5)	
**Primary Language, *n* (%)**				<0.001
English	2870 (77.1)	1510 (80.7)	1360 (73.4)	*h* = 0.17
French	666 (17.9)	293 (15.7)	373 (20.1)	*h* = 0.12
Other	188 (5.0)	68 (3.6)	120 (6.5)	*h* = 0.13
**Living in Canada more than 10 years, *n* (%)**				<0.001
Yes	3457 (92.8)	1768 (94.5)	1689 (91.1)	*h* = 0.13
No	267 (7.2)	103 (5.5)	164 (8.9)	
**Completed post-secondary education, *n* (%)**				0.092
Yes	2697 (72.4)	1378 (73.7)	1319 (71.2)	–
No	1027 (27.6)	493 (26.3)	534 (28.8)	
**Gender identity, *n* (%)**				0.010
Female/woman	3676 (98.7)	1855 (99.1)	1821 (98.3)	*h* = 0.08
Gender diverse ^5^	48 (1.3)	16 (0.9)	32 (1.7)	
**Sexual Orientation, *n* (%)**				<0.001
Heterosexual	3302 (88.7)	1698 (90.8)	1604 (86.6)	*h* = 0.13
Bisexual	218 (5.9)	99 (5.3)	119 (6.4)	ns
Other ^6^	204 (5.5)	74 (4.0)	130 (7.0)	*h* = 0.14
**Relationship/marital status, *n* (%)**				<0.001
In a relationship	2414 (64.8)	1329 (71.0)	1085 (58.6)	*h* = 0.26
Single	1310 (35.2)	542 (29.0)	768 (41.4)	
**Household income (CAD), *n* (%)**				<0.001
39,999 or less	852 (22.9)	343 (18.3)	509 (27.5)	*h* = 0.22
Between 40,000 and 79,999	1243 (33.4)	643 (34.4)	600 (32.4)	ns
80,000 or more	1495 (40.1)	830 (44.4)	665 (35.9)	*h* = 0.17
Prefer not to answer	134 (3.6)	55 (2.9)	79 (4.3)	*h* = 0.07
**Employment status, *n* (%)**				< 0.01
Employed	2333 (62.6)	1220 (65.2)	1113 (60.1)	*h* = 0.11
Not employed	1390 (37.4)	651(34.7)	740 (39.9)	

Note. ^1^ Calculated for adequately screened vs. underscreened; independent samples *t*-tests used for continuous data and chi-square tests of independence for categorical data. For significant differences between categories, we provide Cohen’s *d* (for continuous data) and Cohen’s *h* (for proportions); ns denotes that there is not a significant difference at *p* < 0.05. ^2^ Western and Territories: British Columbia, Alberta, Manitoba, Saskatchewan, Northwest Territories, the Yukon, and Nunavut; Atlantic: Nova Scotia, Prince Edward Island, New Brunswick, and Newfoundland and Labrador. ^3^ Ethnicity was measured using the 2016 categories defined by Statistics Canada [23]. ^4^ The categories *Caribbean*, *Latin, Central and South American*, *African*, *Oceania*, and *Mixed* were recategorized into *Other* due to small individual case counts, alongside those providing otherwise uncategorizable open-ended responses. ^5^ The categories *Male*, *Trans Man*, *Trans Female*, *Non-Binary*, and *Prefer not to answer* were recategorized into *Other* due to small case counts, alongside those providing otherwise uncategorizable open-ended responses. ^6^ The categories *Gay*, *Lesbian*, *Queer*, *Two-Spirit*, and *Prefer not to answer* were recategorized into *Other* due to small individual case counts, alongside those providing otherwise uncategorizable open-ended responses.

## Data Availability

The data sets used for this study will not be published in a publicly available repository in accordance with the ethics proposal approved by the overseeing research ethics board. They will be available from the senior author (ZR) upon reasonable request and upon agreement of confidentiality and data use policies provisioned by the primary institution.

## Supplementary Materials

The following supporting information can be downloaded at: https://www.mdpi.com/article/10.3390/curroncol30070512/s1 Table S1: Detailed Results Tables.
