# Supplementary material for: Are Canadian Women Prepared for the Transition to Primary HPV Testing in Cervical Screening? A National Survey of Knowledge, Attitudes, and Beliefs
Source: Curr Oncol. 2023 Jul 24;30(7):7055–72. doi: 10.3390/curroncol30070512 (PMC10378227; doi:10.3390/curroncol30070512)
Supplement: Supplementary file 1 [file curroncol-30-00512-s001.zip › curroncol-2489355-supplementary.pdf]

Supplementary Material S1: Detailed Results Tables

Cervical Cancer Knowledge Scale									
Item	Adequately Screened (n = 1853)			Underscreened (n = 1871)					
	Proportion of Correct Responses	Lower CI (95%)	Upper CI (95%)	Proportion of Correct Responses	Lower CI (95%)	Upper CI (95%)	χ^2	*p*	Cohen's *h*
The Pap test can detect abnormal cells of the cervix before they become cancer	0.932	0.920	0.944	0.867	0.851	0.882	44.715	<.001	0.22
A woman is at lower risk for developing cervical cancer if she smokes	0.869	0.852	0.884	0.795	0.776	0.814	41.579	<.001	0.20
Discomfort or pain during sex can be a sign of cervical cancer	0.563	0.540	0.585	0.512	0.490	0.535	13.788	.002	0.10
Vaginal bleeding between periods can be a sign of cervical cancer	0.561	0.538	0.584	0.491	0.468	0.514	20.591	<.001	0.14
Vaginal bleeding after menopause can be a sign of cervical cancer	0.546	0.522	0.570	0.502	0.479	0.527	9.074	.008	-
A woman is at higher risk of developing cervical cancer if she has had 5+ sexual partners	0.508	0.484	0.530	0.444	0.421	0.466	15.359	<.001	0.13
Persistent vaginal discharge that smells unpleasant can be a sign of cervical cancer	0.501	0.477	0.523	0.447	0.426	0.471	13.996	.001	0.11
Vaginal bleeding during or after sex can be a sign of cervical cancer	0.490	0.468	0.513	0.407	0.386	0.430	26.401	<.001	0.17

HPV Testing Knowledge Scale									
Item	Adequately Screened (n = 1853)			Underscreened (n = 1871)					
	Proportion of Correct Responses	Lower CI (95%)	Upper CI (95%)	Proportion of Correct Responses	Lower CI (95%)	Upper CI (95%)	χ^2	*p*	Cohen's *h*
If the HPV test shows a woman has HPV, this means she needs further follow-up	0.893	0.879	0.906	0.849	0.832	0.865	16.523	<.001	0.13
If the HPV test shows that a woman has HPV, this means she is at increased risk for cervical cancer	0.786	0.768	0.805	0.729	0.708	0.750	19.437	<.001	0.13
Women who have received the HPV vaccine do not need the HPV test	0.711	0.691	0.732	0.649	0.628	0.671	16.262	<.001	0.13
If the HPV test shows a woman has HPV, this means she already has cervical cancer	0.683	0.660	0.704	0.617	0.595	0.638	18.469	<.001	0.14
An HPV test can be done at the same time as a Pap test	0.597	0.575	0.617	0.547	0.525	0.568	11.265	.002	0.10
An HPV test can tell a woman how long she has had HPV	0.377	0.354	0.399	0.318	0.297	0.340	14.000	<.001	0.12
If HPV is found during HPV testing, this is the same thing as an abnormal Pap test result	0.251	0.230	0.271	0.232	0.212	0.250	12.465	.172	-
The HPV test sample can be collected by the woman herself using a specialized HPV self-sampling kit	0.198	0.180	0.217	0.210	0.193	0.229	2.904	.357	-

HPV General Knowledge Scale									
Item	Adequately Screened (n = 1853)			Underscreened (n = 1871)					
	Proportion of Correct Responses	Lower CI (95%)	Upper CI (95%)	Proportion of Correct Responses	Lower CI (95%)	Upper CI (95%)	χ^2	*p*	Cohen's *h*
HPV can be passed on during sexual intercourse	0.848	0.832	0.864	0.796	0.778	0.814	18.121	<.001	0.14
Using condoms reduces the chances of HPV transmission	0.829	0.813	0.846	0.779	0.760	0.797	16.577	<.001	0.13
A person could have HPV for many years without knowing it	0.818	0.800	0.837	0.774	0.756	0.794	11.146	.001	0.11
Having many sexual partners increases the risk of getting HPV	0.823	0.805	0.841	0.761	0.743	0.782	22.655	<.001	0.15
HPV can cause cervical cancer	0.816	0.797	0.833	0.747	0.727	0.767	26.557	<.001	0.17
HPV is very rare	0.763	0.743	0.783	0.697	0.675	0.718	21.021	<.001	0.15
A person with no symptoms cannot transmit the HPV infection	0.700	0.679	0.721	0.663	0.640	0.685	15.357	.016	-
Men cannot get HPV	0.675	0.653	0.695	0.597	0.576	0.618	25.199	<.001	0.16
HPV always has visible signs and symptoms	0.647	0.625	0.669	0.602	0.580	0.626	8.308	.005	-
HPV can be transmitted through genital skin-to-skin contact	0.561	0.538	0.584	0.516	0.493	0.539	8.334	.006	-
There are many types of HPV	0.495	0.474	0.516	0.492	0.469	0.513	7.132	.816	-
HPV can be transmitted through anal sex	0.498	0.475	0.522	0.453	0.430	0.478	8.402	.006	-
HPV can cause HIV/AIDS	0.499	0.476	0.521	0.432	0.411	0.454	16.990	<.001	0.13
HPV can be transmitted through oral sex	0.484	0.459	0.507	0.439	0.414	0.460	8.999	.005	-

HPV can cause genital warts	0.476	0.452	0.498	0.437	0.416	0.459	9.704	.017	-
Having sex at an early age increases the risk of getting HPV	0.460	0.437	0.483	0.399	0.376	0.420	14.826	<.001	0.12
HPV can be cured with antibiotics	0.368	0.344	0.391	0.323	0.303	0.346	8.536	.004	-
HPV is a bacterial infection	0.334	0.310	0.355	0.302	0.281	0.323	4.344	.040	-
HPV can cause oral cancer	0.320	0.300	0.342	0.261	0.241	0.281	17.468	<.001	-
HPV infections always lead to health problems	0.294	0.274	0.315	0.242	0.223	0.262	12.775	<.001	0.12
HPV can cause anal cancer	0.283	0.264	0.305	0.241	0.220	0.260	8.521	.004	-
Most sexually active people will get HPV at some point in their lives	0.252	0.232	0.272	0.222	0.202	0.241	18.762	.032	-
HPV can cause cancer of the penis	0.251	0.231	0.270	0.203	0.184	0.222	12.614	.001	0.11

HPV Testing Attitudes and Beliefs Scale									
I feel that…	Adequately Screened (n = 1853)			Underscreened (n = 1871)					
	M (SD) [out of 7]	Lower CI (95%)	Upper CI (95%)	M (SD) [out of 7]	Lower CI (95%)	Upper CI (95%)	*t*	*p*	Cohen's *d*
**Personal Barriers**									
the HPV test would be painful	3.226 (1.524)	3.157	3.296	3.752 (1.497)	3.683	3.825	10.639	<.001	0.35
I would be embarrassed to show my genitals to a healthcare professional during the HPV test	2.679 (1.721)	2.606	2.754	3.781 (1.962)	3.695	3.868	18.218	<.001	0.60
I have other priorities more important than having the HPV test	2.773 (1.468)	2.710	2.833	3.465 (1.581)	3.401	3.540	13.848	<.001	0.45
going to see a healthcare professional to have the HPV test would take too much time	2.628 (1.511)	2.559	2.695	3.370 (1.629)	3.295	3.439	14.406	<.001	0.47
I would be embarrassed to get tested for HPV because it is a sexually transmitted infection	2.570 (1.615)	2.503	2.638	3.016 (1.688)	2.939	3.098	8.236	<.001	0.27
I would not need to have the HPV test because I do not have symptoms	2.231 (1.304)	2.175	2.292	2.704 (1.451)	2.638	2.771	10.474	<.001	0.34
healthcare professionals doing the HPV test would be rude to me	2.089 (1.314)	2.030	2.150	2.509 (1.455)	2.443	2.582	9.251	<.001	0.30
**Social Norms**									
my partner's opinion about getting the HPV test would be important to me	3.710 (1.993)	3.617	3.797	3.862 (1.885)	3.784	3.949	2.400	.064	-
my family's opinion about getting the HPV test would be important to me	3.151 (1.852)	3.065	3.231	3.325 (1.799)	3.248	3.409	2.911	.004	0.10
my friends' opinion about getting the HPV	2.919 (1.722)	2.845	2.997	3.084 (1.690)	3.008	3.165	2.948	.003	0.10

HPV Testing Attitudes and Beliefs Scale									
I feel that…	Adequately Screened (n = 1853)			Underscreened (n = 1871)					
	M (SD) [out of 7]	Lower CI (95%)	Upper CI (95%)	M (SD) [out of 7]	Lower CI (95%)	Upper CI (95%)	*t*	*p*	Cohen's *d*
test would be important to me									
opinions I see on social media about getting the HPV test would be important to me	2.761 (1.733)	2.686	2.839	2.988 (1.715)	2.909	3.072	4.027	<.001	0.13
Confidence									
having the HPV test would be a good way to identify problems before they become cancer	6.176 (0.998)	6.131	6.221	5.987 (1.068)	5.939	6.031	5.605	<.001	0.18
if the HPV test showed I have HPV, it is important to follow up on it	6.446 (0.930)	6.405	6.487	6.288 (0.967)	6.243	6.328	5.086	<.001	0.17
if HPV+, I feel that I would need more information to help me deal with the results	5.937 (1.159)	5.883	5.993	5.918 (1.132)	5.867	5.967	0.519	0.604	-
the HPV test would be safe	5.983 (0.984)	5.940	6.029	5.695 (1.109)	5.646	5.744	8.377	<.001	0.27
my healthcare professional's opinion about getting the HPV test would be important to me	5.650 (1.308)	5.591	5.709	5.297 (1.407)	5.226	5.357	7.930	<.001	0.26
public health agencies' opinions about getting the HPV test would be important to me	5.035 (1.542)	4.964	5.108	4.816 (1.528)	4.747	4.888	4.348	<.001	0.14
Worries									
I would be worried about starting screening with the HPV test at 30 instead of 21	4.285 (1.814)	4.205	4.363	4.089 (1.684)	4.007	4.165	3.423	.001	0.11

HPV Testing Attitudes and Beliefs Scale									
I feel that…	Adequately Screened (n = 1853)			Underscreened (n = 1871)					
	M (SD) [out of 7]	Lower CI (95%)	Upper CI (95%)	M (SD) [out of 7]	Lower CI (95%)	Upper CI (95%)	*t*	*p*	Cohen's *d*
I would be worried about getting tested with the HPV test less often than every 3 years	3.868 (1.661)	3.795	3.941	3.574 (1.520)	3.500	3.639	5.628	<.001	0.18
I would be worried about starting screening with the HPV test at 25 instead of 21	3.747 (1.740)	3.668	3.824	3.676 (1.550)	3.604	3.750	1.314	.189	-

HPV Self-Sampling Attitudes and Beliefs Scale									
I feel that…	Adequately Screened (n = 1853)			Underscreened (n = 1871)					
	M (SD) [out of 7]	Lower CI (95%)	Upper CI (95%)	M (SD) [out of 7]	Lower CI (95%)	Upper CI (95%)	*t*	*p*	Cohen's *d*
Concerns									
if I did HPV self-sampling, I would worry that I am not doing it right	4.740 (1.721)	4.663	4.817	4.573 (1.727)	4.493	4.648	2.968	.003	0.10
if I did HPV self-sampling, I could harm myself	2.873 (1.546)	2.802	2.943	3.041 (1.585)	2.968	3.112	3.278	.001	0.11
if I did HPV self-sampling, I could get an infection	2.483 (1.375)	2.428	2.545	2.640 (1.348)	2.574	2.698	3.517	<.001	0.12
I would feel embarrassed doing HPV self-sampling	2.388 (1.539)	2.320	2.457	2.496 (1.510)	2.427	2.564	2.171	.030	-
Autonomy									
if I did HPV self-sampling, I would be more in control of my body	4.775 (1.485)	4.710	4.838	5.173 (1.640)	5.112	5.240	8.404	<.001	0.28
I would prefer doing HPV self-sampling at home because it would save me travelling to see a healthcare professional	4.678 (1.775)	4.594	4.752	5.175 (1.401)	5.101	5.247	8.864	<.001	0.29
I would be more comfortable doing the swab by myself using HPV self-sampling than having an HPV test done by a healthcare professional	4.146 (1.817)	4.055	4.224	4.909 (1.735)	4.834	4.992	13.092	<.001	0.43
